# Diversity of the microbial community and cultivable protease-producing bacteria in the sediments of the Bohai Sea, Yellow Sea and South China Sea

**DOI:** 10.1371/journal.pone.0215328

**Published:** 2019-04-11

**Authors:** Jiang Zhang, Ming Chen, Jiafeng Huang, Xinwu Guo, Yanjiao Zhang, Dan Liu, Ribang Wu, Hailun He, Jun Wang

**Affiliations:** 1 School of Life Science, Central South University, Changsha, China; 2 Sanway Gene Technology Inc., Changsha, China; 3 Shandong Province Key Laboratory of Applied Mycology, School of Life Sciences, Qingdao Agricultural University, Qingdao, China; Universite Paris-Sud, FRANCE

## Abstract

The nitrogen (N) cycle is closely related to the stability of marine ecosystems. Microbial communities have been directly linked to marine N-cycling processes. However, systematic research on the bacterial community composition and diversity involved in N cycles in different seas is lacking. In this study, microbial diversity in the Bohai Sea (BHS), Yellow Sea (YS) and South China Sea (SCS) was surveyed by targeting the hypervariable V4 regions of the 16S rRNA gene using next-generation sequencing (NGS) technology. A total of 2,505,721 clean reads and 15,307 operational taxonomic units (OTUs) were obtained from 86 sediment samples from the three studied China seas. LEfSe analysis demonstrated that the SCS had more abundant microbial taxa than the BHS and YS. Diversity indices demonstrated that Proteobacteria and Planctomycetes were the dominant phyla in all three China seas. Canonical correspondence analysis (CCA) indicated that pH (*P* = 0.034) was the principal determining factors, while the organic matter content, depth and temperature had a minor correlated with the variations in sedimentary microbial community distribution. Cluster and functional analyses of microbial communities showed that chemoheterotrophic and aerobic chemoheterotrophic microorganisms widely exist in these three seas. Further research found that the cultivable protease-producing bacteria were mainly affiliated with the phyla Proteobacteria, Firmicutes and Bacteroidetes. It was very clear that Pseudoalteromonadaceae possessed the highest relative abundance in the three sea areas. The predominant protease-producing genera were *Pseudoalteromonas* and *Bacillus*. These results shed light on the differences in bacterial community composition, especially protease-producing bacteria, in these three China seas.

## Introduction

Marine sediment, an important part of the aquatic environment, is a mixture of material deposited on the seafloor that mainly originates from the erosion of continents and the deposition of biological products [1]. Marine sediments are nutrient-rich habitats that harbor diverse microbial communities because of the reservoirs of absorbed nutrients, pesticides, and marine high-molecular-weight organic nitrogen (HMWON) [2, 3]. Subseafloor sediments accumulate large amounts of organic and inorganic materials and contain a highly diverse microbial ecosystem. Sediment microorganisms are very efficient at cycling nutrients, metabolizing foreign compounds in marine ecosystems and colonizing new ecological niches [4]. Among them, heterotrophic bacteria in marine environments are responsible for the degradation of organic biopolymers and the redistribution of organic matter, which have ecological significance in carbon and nitrogen cycling [4]. In recent years, many studies have focused on the microbial diversity of marine sediments. Quaiser et al. reported that the Marmara sediment clustered with the soil metagenome, highlighting the common ecological role of both types of microbial communities in the degradation of organic matter and the completion of biogeochemical cycles [1, 4]. Moreover, Heebok et al. found that the microbial communities in the surface sediments were distinct from those in the subsurface sediments in Jeju Island. Furthermore, this study also provided fundamental information on the potential interactions mediated by microorganisms driving different biogeochemical cycles in coastal sediments [5]. Zeng et al. found that diverse microbial communities inhabit panarctic marine sediments and highlighted the potential roles for Archaea and Bacteria in global biogeochemical cycles in Arctic Kongsfjorden and Sub-Arctic Northern Bering Sea sediments [6]. The nitrogen cycle is an important part of material circulation in the marine ecological system, and many N-cycling processes are microbially mediated [7]. In the marine nitrogen cycle, particulate organic nitrogen (PON) is first decomposed into dissolved inorganic nitrogen (DON) and then ammonified, nitrified, and denitrified, mainly performed by microbial enzymes, especially proteases [8]. Many cultivated bacteria from marine sediments, such as *Pseudomonas*, *Pseudoalteromonas*, *Alkalimonas collagenimarina*, *Colwellia*, *Planococcus*, *Alteromonas*, *Marinobacter*, *Idiomarina*, *Halomonas*, *Vibrio*, *Shewanella* and *Rheinheimera*, have been demonstrated to be protease-producing bacteria [1, 9]. China is a maritime country containing four large offshore seas across the tropical, subtropical and temperate climate zones. There are many untapped microbial resources in the China offshore seas. The China coastal aquaculture is large, and the marine environment has been seriously affected by humans, causing nitrogen enrichment. Thus, the microbial ecosystem, especially protease-producing bacteria of the China offshore seas, has been greatly affected [10]. Research on microbial diversity and protease-producing bacteria in China's offshore seas will aid in the study of environmental pollution in coastal areas and will help to develop the environmental microbial resources in China. Based on pyrosequencing 16S rRNA gene clone libraries, Zheng et al. observed that the sediment median grain size and dissolved oxygen were major factors regulating bacterial community in the sediment of Liaodong Bay [11]. Protease-producing bacteria belonging to *Photobacterium*, *Bacillus*, and *Vibrio* were the richest cultivated protease-producing bacteria in Jiaozhou Bay marine sediments [7]. Research on the East China Sea showed that most bacteria were present in the 3–5 cm or 5–8 cm sediment layers and Proteobacteria, Chloroflexi, and Planctomycetacia were the dominant cultivable bacteria [10].

The BHS and YS are semienclosed continental seas of the northwestern Pacific Ocean in northern China. The physical and biotic environments of these sea regions have shown a strong seasonality in the nutrient components and their concentrations [10, 12]. The SCS, the largest marginal sea in the tropical-subtropical western North Pacific, is characterized by a complicated basin topography and abundant gravity flow sedimentation and ocean dynamics [13]. These three seas are exceptionally complex and dynamic aquatic ecosystems, due to the significant recycling of nutrients and organic matter. To date, systematic research comparing microbial diversity and the organic nitrogen digested by microbial communities in these three marginal seas is lacking. In this study, next-generation sequencing technology was used to investigate the microbial diversity and community composition of marine sediments in three China seas (BHS, YS and SCS), especially at coastal sampling sites; the results provide a distinct view of the microbial compositions in different environmental habitats. Through analyzing the common and unique core bacteria in each China Sea, the ecological roles and coexistence patterns of these bacteria in different marine environments were further explored. These results will help us to understand the functional relationship between microorganisms and their physiological characteristics. Finally, we screened the protease-producing bacteria, which not only contributes to our understanding of the basic rules governing the marine nitrogen cycle but also provides an important theoretical basis for the development and utilization of new marine microbial resources and protection of the marine environment.

## Materials and methods

### Sediment sample collection and environmental factors

**Fig 1:**
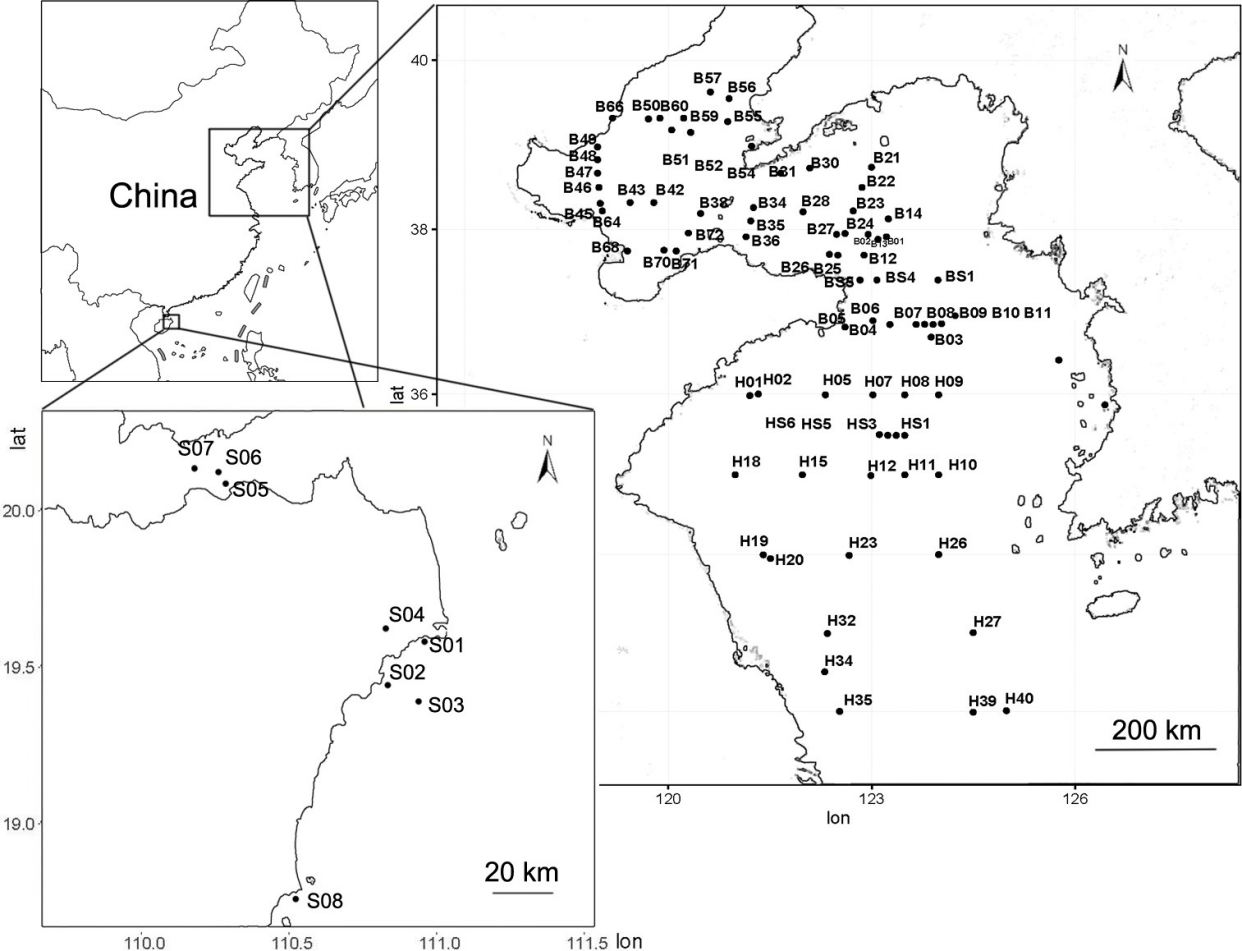
Geographic location of sampling sites in the YS (Yellow Sea), BHS (Bohai Sea) and SCS (South China Sea).

Seventy-eight sediment samples (mud) (Fig 1) were collected from the YS and BHS. The sediment samples from the YS and BHS were collected using a 0.05 m^3^ sterile stainless steel grab sampler (Wildco, Florida, USA) in 2015 aboard the Dongfanghong #2 research vessel. All sampling wares and centrifuge tubes were first treated with moist heat sterilization. Taking different navigation plans into consideration from the YS and the BHS, the coastal sampling points were included from the SCS. Eight sediment samples (sand) (Fig 1) (upper 3 cm) from the SCS were collected. A global positioning system was used to determine the sampling positions. We determined the sample number based on the sampling order. All samples were stored in sterile hermetic bags. A portion of each sample was separated to screen for protease-producing bacteria and to take environmental measurements immediately after sampling; the remainder of each simple was stored at -20°C until DNA extraction was conducted in our laboratory. Sampling sites did not involve endangered or protected species and the sediments were collected during the oceanological comprehensive scientific investigation organized by the National Nature Science Foundation of China (41349901).

Seven environmental factors, including depth, salinity, temperature, pH, organic matter (OM), water and total phosphorus (TP) and total nitrogen (TN) were selected for this study. The salinity, temperature and depth were assessed by casting a SeaBird CTD system. The measurements of OM, TP and TN were in compliance with the national environmental protection standards of the People’s Republic of China published in 2012 [14]. Samples were preprocessed by adding triple volumes of sterile water (v/v) to each sediment sample and then shaking the mixture for 30 min at 25°C. A filtrate was obtained using double filter papers. The OM content was determined by potassium bichromate titrimetric method; the TP content was determined by ammonium molybdate spectrophotometric method [15]; the TN content was determined by measuring the potassium persulfate oxidation via an ultraviolet spectrometer [16]. The pH level was detected using a pH meter (Ohaus, New Jersey, USA).

### DNA extraction and PCR

Total genomic DNA was extracted from 1 g of the marine sediment samples using E.A.N.A. Soil DNA kit (OMEGA, Georgia, GA, USA) following the manufacturer’s instructions. The V4 region of the 16S rRNA gene was amplified with polymerase chain reaction (PCR) using the primers 515F (5’-GTGCCAGCMGCCGCGGTAA-3’) and 806R (5’-GGACTACHVGGGTWTCTAAT-3’)[17].

### Sequencing and data analysis

16S rRNA gene libraries were sequenced using an Illumina MiSeq (San Diego, CA, USA) platform and the sequencing data were base called and demultiplexed using MiSeq Reporter v.1.8.1 (Illumina, San Diego, CA, USA) with the default parameters. The OTUs were selected at 97% similarity. The richness estimators (ACE and Chao) and diversity indices (Shannon and Simpson) were calculated using the Mothur program. OTU comparisons were performed using the Venn diagram package. Boxplots were used to compare the microbial diversity of the different groups. A neighbor-joining phylogenetic tree was used to investigate the similarity of species abundance using the unweighted pair group with arithmetic mean (UPGMA) clustering [18]. Using a relative abundance matrix, LEfSe (the linear discriminant analysis coupled with effect size measurements method) analysis was performed using the Kruskal-Wallis rank sum test to detect the microbial taxa with significantly different abundances between the three sea areas and using LDA to estimate the effect size of each taxon [19]. All tests for significance were two-sided, and *P* values < 0.05 were considered statistically significant. Dot plots were generated to compare the microbial relative abundances in the different groups. The significance of the separation among groups from the same sea was determined using an analysis of similarity (ANOSIM) test. This test is a generalization of the univariate ANOVA and it has the ability to consider all variables during the calculation of similarity among populations based on the Euclidean distance matrix. The relationship between the microbial diversities and environmental factors was implemented using canonical correspondence analysis (CCA). The Functional Annotation of Prokaryotic Taxa (FAPROTAX) pipeline was used to predict the functional potential of prokaryotic communities [20, 21]. The complete database for the FAPROTAX includes over 7600 annotations and covers over 4600 taxa, and is available at www.zoology.ubc.ca/louca/FAPROTAX. Louca et al. provide a detailed evaluation of FAPROTAX, including a direct comparison with metagenomics [20]. All statistical analyses were carried out using R software (http://www.cran.r-project.org). The raw sequences generated in the present study were deposited in the Genome Sequence Archive in the BIG Data Center (BIG Data Center Members 2017), Beijing Institute of Genomics (BIG), Chinese Academy of Sciences, under accession number CRA000634. This information is publicly accessible at *http*:*//bigd*.*big*.*ac*.*cn/gsa*.

### Screening and identification of protease-producing bacteria

The sediment samples were screened for protease-producing bacteria using the dilution-plate method on a screening medium containing 0.5% peptone, 0.1% yeast, 1% milk, 1.5% agar and artificial sea water (pH 7.8). Artificial sea water (ASW) was composed of 2.8% sodium chloride, 0.7% magnesium sulfate heptahydrate, 0.1% potassium chloride, 0.6% magnesium chloride hexahydrate, 0.1% calcium chloride and distilled water. In brief, 1 g (wet weight) of sediment sample was 10-fold serially diluted to a 10^−5^ dilution with ASW. Aliquots of 100 μl of the diluted samples (10^−1^–10^−5^ dilution) were spread on the screening medium and incubated at 18°C until colonies with clear hydrolysis zones (transparent ring with radius bigger than 1 mm) were visible. Morphologically different colonies with hydrolytic zones were purified by repeated streaking on the same medium. The purified strains were stored in 50% (v/v) glycerol at -80°C.

Genomic DNA of the protease-producing bacteria were extracted using a bacterial genomic DNA Extraction kit (Biospin, China). The 16S rDNA was PCR-amplified with the forward primer 27F (5’-AGAGTTTGATCMTGGCTCAG-3’) and the reverse primer 1492R (5′-GGTTACCTTGTTACGACTT-3′). The sequences generated in this study were identified by searching for the most similar sequences in the NCBI GenBank using BLASTn.

## Results

### Diversity and composition of microbial communities in the BHS, YS and SCS

**Fig 2:**
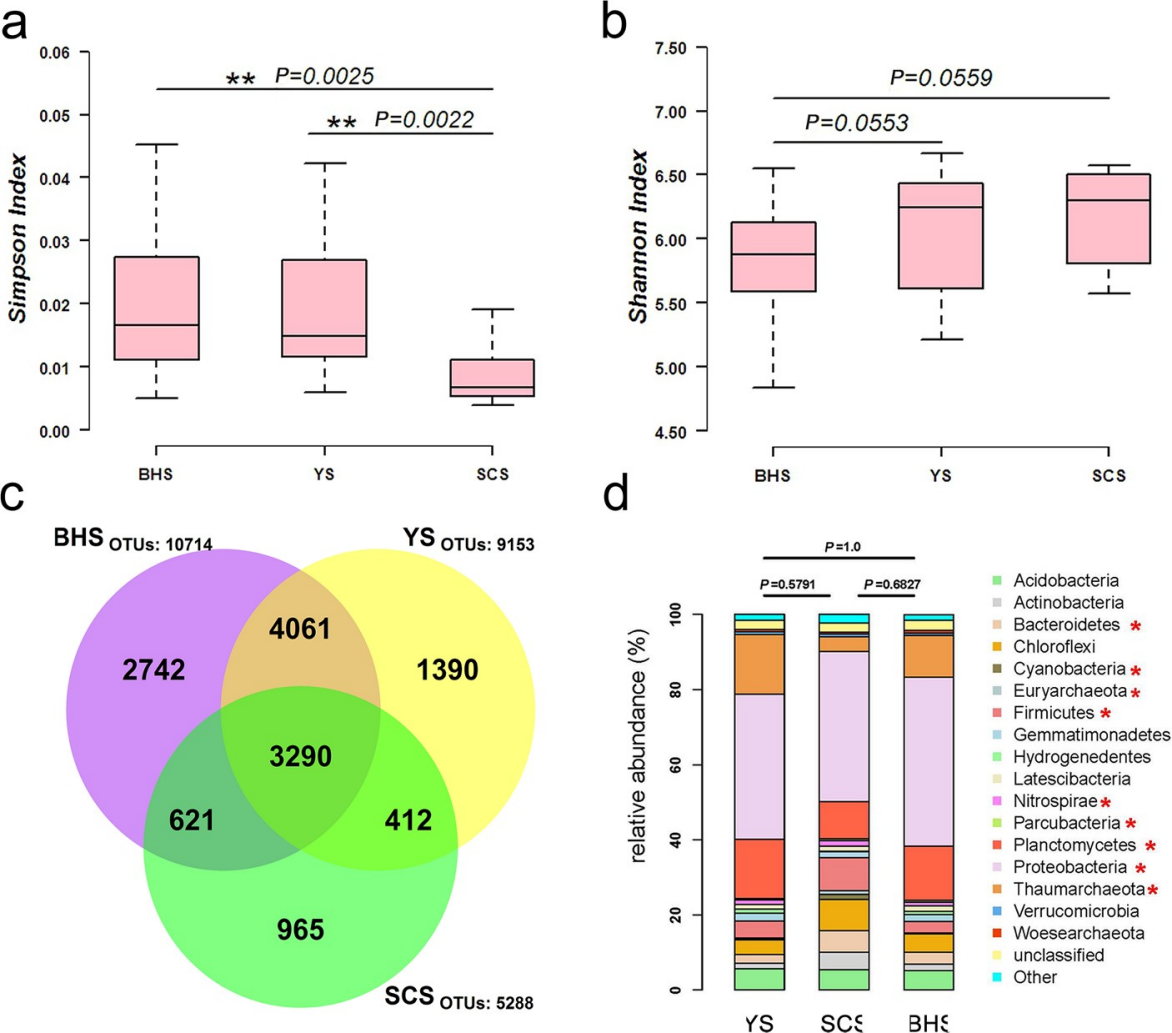
**(a) Boxplot of Simpson indices calculated using the OTU data in the three sea areas. (b) Boxplot of Shannon indices calculated using the OTU data in the three sea areas. (c) Venn diagram denoting the number of unique and shared species in the different libraries at the 3% distance level.** The yellow circle indicates the YS, the purple circle indicates the BHS, and the green circle indicates the SCS. **(d) Diagram showing the relative abundance of bacterial composition in the three sea areas at the phylum level.** The red star represents the specific bacterial taxa showing significant differences in one of the three groups.

Next-generation sequencing with the 16S rRNA amplicons was performed on 86 different sediment samples collected from three China seas (BHS, YS and SCS)(Fig 1). A total of 13,481 OTUs remained after resampling. The numbers of OTUs in the BHS, YS and SCS were 10,714, 9,153 and 5,288 OTUs, respectively (Fig 2C). Based on the Simpson index and Shannon index, the microbial diversity in the SCS was significantly higher than that in the YS (*P* < 0.01) and BHS (*P* < 0.01) (Fig 2A and 2B).

Based on alignment with the SILVA database, 13,481 OTUs were identified at different levels of taxonomic precision, and they spanned 59 phyla, 128 classes, 221 orders, 347 families and 517 genera. At the phylum level, the three China seas possessed a similar microbial community structure, with Acidobacteria, Actinobacteria, Bacteroidetes, Chloroflexi, Firmicutes, Gemmatimonadetes, Latescibacteria, Planctomycetes, Proteobacteria and Thaumarchaeota making up the top ten ubiquitous phyla. Among these ten phyla, Proteobacteria (39.32%-45.54%) and Planctomycete (10.12%-15.83%) showed the two highest relative abundances in the three sea areas (Fig 2D).

### Microbial groups with statistical differences

**Fig 3:**
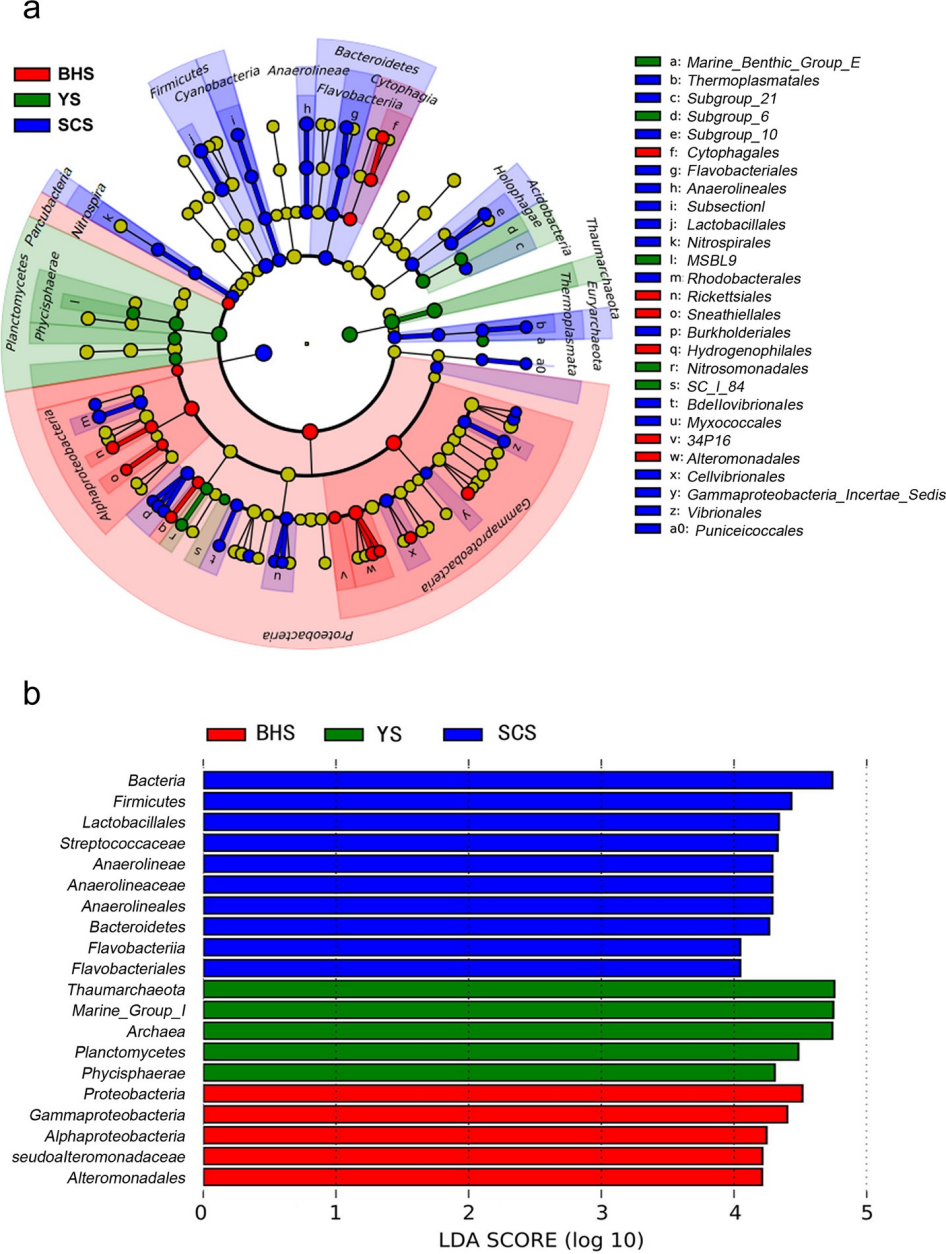
Taxonomic cladogram produced from LEfSe analysis. (A) Cladogram representing statistically significant differences in bacterial clades among the three sea areas. Small circles shaded with different colors in the diagram represent abundances of those taxa in the respective group (red, BHS; green, YS; blue, SCS; and yellow, nonsignificant). Each circle’s diameter is proportional to the taxon’s abundance. (B) Indicator of bacterial groups within the three seas of sediments with LDA score ≥ 4.

To determine which classified microbial taxa had significant differences in abundance among the three China seas, LEfSe, a metagenomic biomarker discovery approach was used [22]. As shown in Fig 3A, 45 microbial clades presented statistically significant differences with an LDA threshold ≥ 2. Taking statistically significant and biologically consistent differences in account, the resultant cladograms showed taxa with LDA values higher than four for clarity (Fig 3B). There were ten microbial taxa enriched in the SCS, five microbial taxa enriched in the YS, and five microbial taxa enriched in the BHS. The most differentially abundant microbial taxa in SCS were Bacteria (the kingdom), Bacteroidetes (the phylum), Firmicutes (the phylum), Flavobacteriia (the class), Anaerolineae (the class), Flavobacteriales (the order), Anaerolineae (the order), Lactobacillales (the order), Anaerolineaceae (the family) and Streptococcaceae (the family). Meanwhile, Proteobacteria (the phylum), Alphaproteobacteria (the class), Gammaproteobacteria (the class), Alteromonadales (the order) and Pseudoalteromonadaceae (the family) were significantly enriched in the BHS. Archaea (the kingdom), Thaumarchaeota (the phylum), Planctomycetes (the phylum), Marine Group I (the class) and Phycisphaerae (the class) were the representative taxa in the YS.

### Microbial communities in the offshore samples from the BHS, YS and SCS

**Fig 4:**
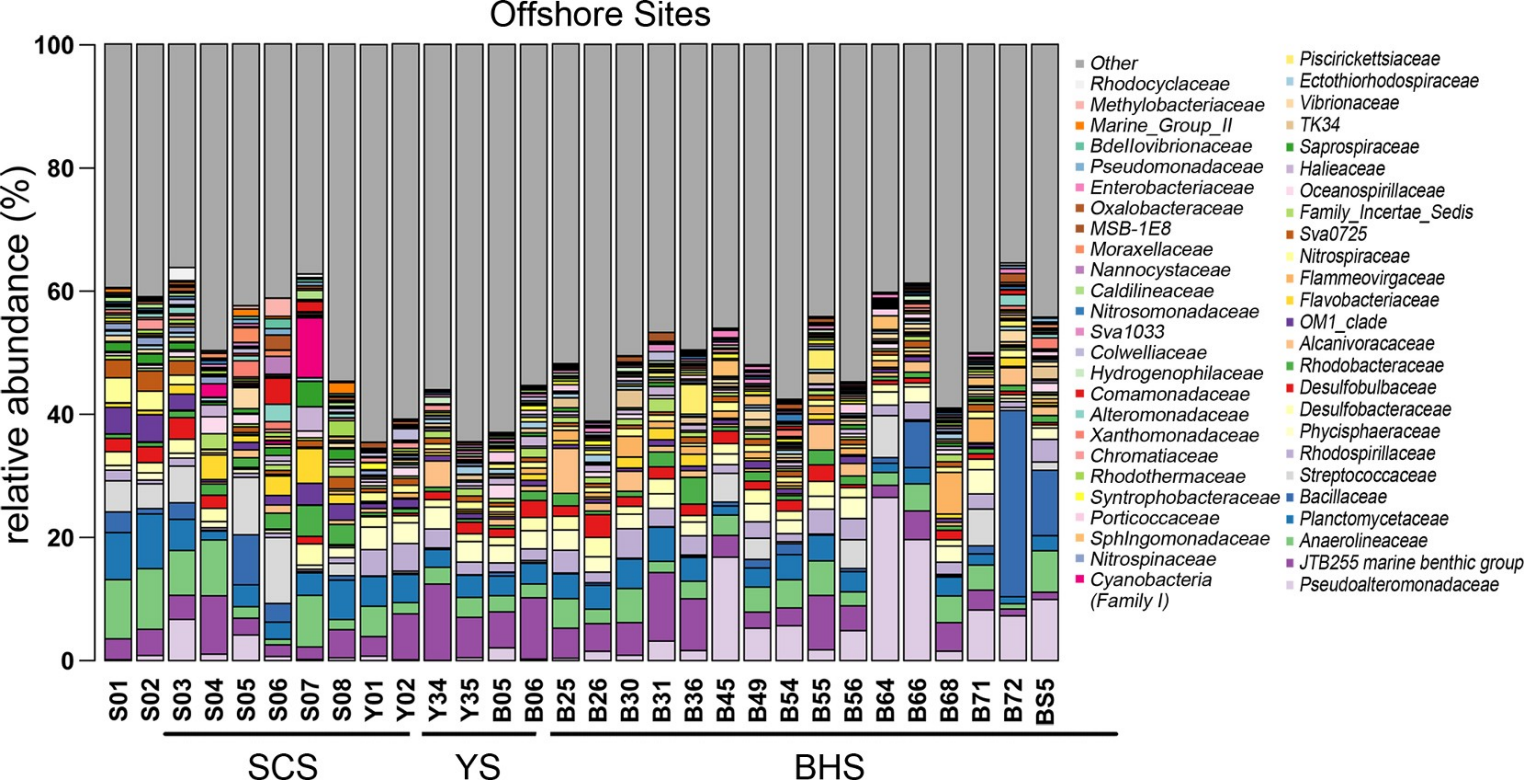
Histogram method for determining the relative abundance of microorganisms at different inshore sampling sites in the three China seas at the family level.

As showed in Fig 4, in the SCS, Pseudoalteromonadaceae was highly represented in S03 and S05 and JTB255 marine benthic group and Anaerolineaceae in S01, S02, S04 and S07. Except in S04, Planctomycetaceae was common in the SCS. In addition, Desulfobulbaceae was present only in small proportions in S05 and S08. Streptococcaceae was highly represented in S01, S02, S03, S05 and S06. In particular, Cyanobacteria (Family I) was proportionally dominant in S07, followed by S04. In the YS, Y01, Y02, Y34 and Y35 had similar microbial compositions, including JTB255 marine benthic group, Anaerolineaceae, Planctomycetaceae, Rhodospirillaceae and Phycisphaeraceae, and all these families accounted for a large proportion of the bacteria in the YS. Moreover, Sphingomonadaceae appeared more in Y34, and Desulfobulbaceae occupied some proportions of the Y34 and Y35 samples. In the BHS, B45, B64, B66, B68, B71, B72 and BS5 had different microbial structures from the other offshore sampling sites. Pseudoalteromonadaceae was proportionally higher in B45, B64 and B66, B72 and BS5 had a large proportion of Bacillaceae, while B66, B30, B68 and B71 had more Alcanivoracaceae. At the family level, Planctomycetaceae and Anaerolineaceae were more abundant in the SCS than the YS and BHS. JTB255 marine benthic group was most abundant in the YS and Pseudoalteromonadaceae in BHS. Proteobacteria (especially the classes Alphaproteobacteria and Gammaproteobacteria) are the most important component of the offshore microbiome. Furthermore, each offshore sea sample has a large number of unknown species especially the YS, which provides new information relevant to the discovery of potential new microbial species (Fig 4).

### Functional analysis of marine sediment microbial communities

**Fig 5:**
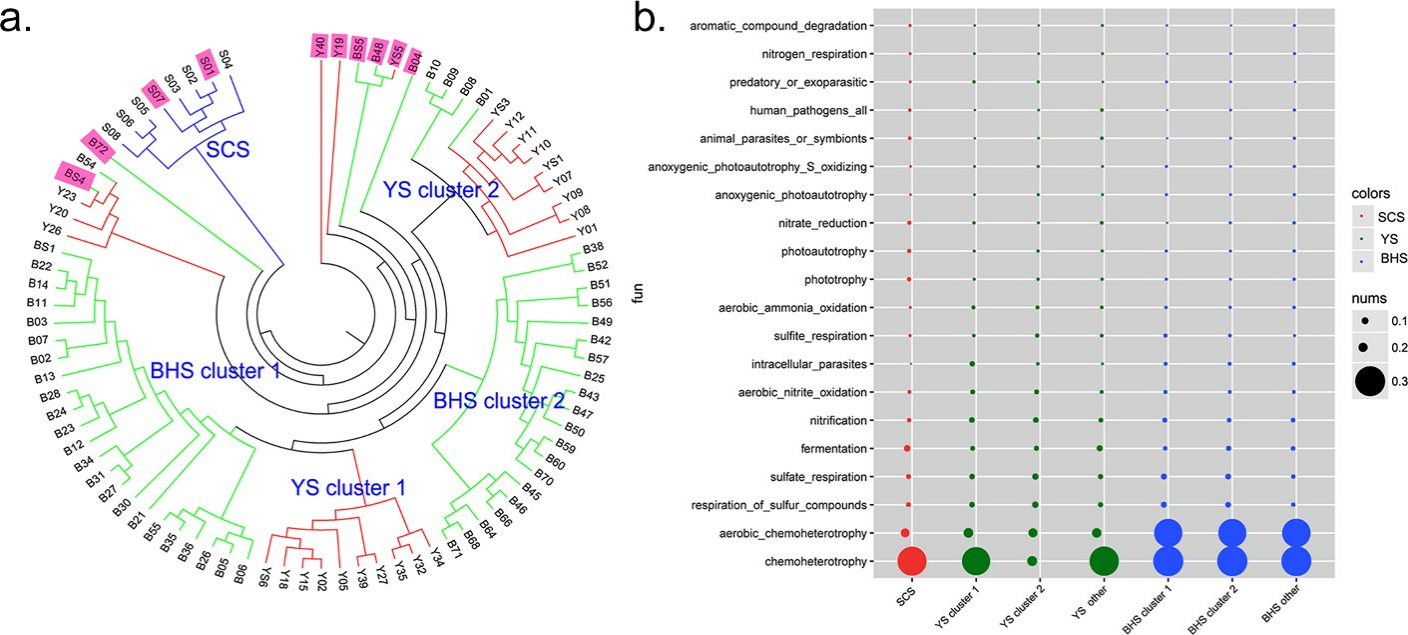
**(a) Cluster analysis showing the similarity of bacterial communities recovered from 86 sampling sites in the SCS, YS and SCS based on the OTU numbers of each sampling station.** Eighty-six sampling sites could be classified into seven clusters. **(b) Functional analysis of microbial communities.** Bubbleplot representing the functional microorganisms in the seven clusters from the three China Seas.

As shown in Fig 5A, cluster analysis revealed a conservation of the community composition similarity among different marine sediments according to the OTUs of each sampling site. A total of 3 clusters (Fig 5A, clusters I, II and III) were observed from the 86 sediment samples in the three sea areas. All samples from the SCS were more similar to each other and clustered separately from the BHS and YS clusters (Fig 5A, cluster I). In the BHS, one sampling site (B72) was clustered separately (cluster II), and the others in the BHS and all sampling sites in the YS were clustered into the third group (cluster III). The functional microorganism analysis of seven clusters(Fig 5B) showed that the chemoheterotrophic and aerobic chemoheterotrophy microorganisms widely existed in the three China seas, especially in the BHS. The microorganisms that participated in the sulfur cycle process were ubiquitous in the oceans. In addition, a large number of microorganisms were also involved in the nitrogen cycle, such as nitrification, aerobic nitrite oxidation, nitrate reduction and nitrogen respiration. More interestingly, the YS cluster II had fewer chemoheterotrophic and aerobic chemoheterotrophy microorganisms (Fig 5B).

**Fig 6:**
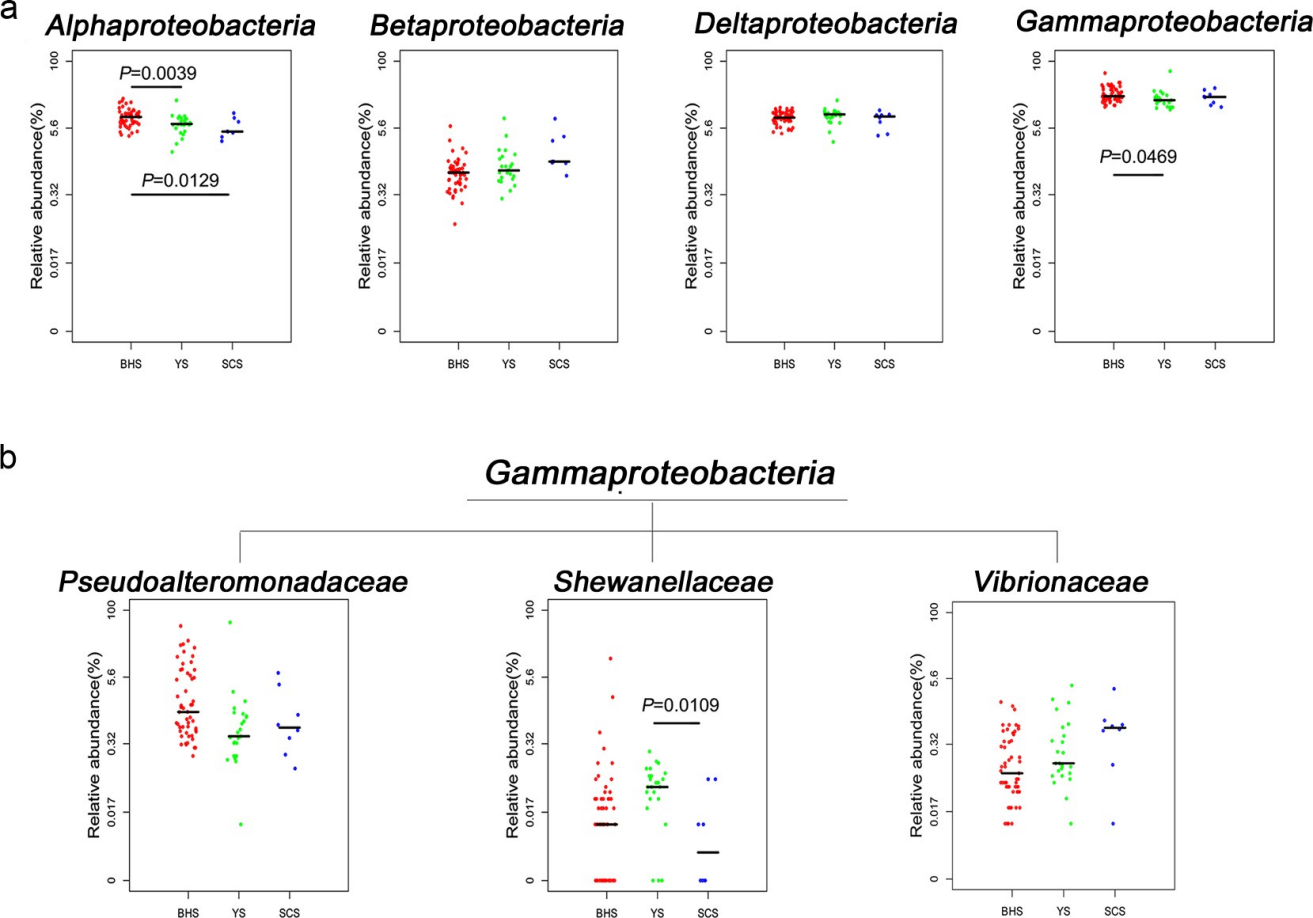
**(a) Dot plots of relative abundance.** The dot plots show the relative abundance of the four classes of Proteobacteria. **(b) Dot plots of the relative abundances of the four genera of Gammaproteobacteria in the three seas.** The bar represents the median.

The components of protease-producing marine microorganisms were further studied and the phylum Proteobacteria and its four major classes Alphaproteobacteria, Betaproteobacteria, Deltaproteobacteria and Gammaproteobacteria were detected in all the sediment samples (Fig 6A). Alphaproteobacteria and Gammaproteobacteria were more common in the BHS, accounting for 9.40% and 25.24% relative abundance respectively. Betaproteobacteria with 2.68% relative abundance in the SCS and Deltaproteobacteria with 9.80% relative abundance in YS were both present in slightly higher proportions than in other seas (Fig 6A). In addition, the Gammaproteobacteria family mainly included Vibrionaceae, Pseudoalteromonadaceae, Shewanellaceae and Porticoccaceae, but the current related studies showed that only Vibrionaceae, Pseudoalteromonadaceae and Shewanellaceae were involved in protease production processes (Fig 6B). Pseudoalteromonadaceae possessed the highest relative abundance in three sea areas (5.48% in BHS, 3.03% in YS and 1.74% in SCS, Fig 6B). The other family, Shewanellaceae, accounted for 0.07% of the relative abundances in the YS samples, which was higher than that in the SCS and BHS samples. The third family Vibrionaceae showed a higher relative abundance with a 0.15% relative abundance in the SCS samples, a value greater than that found in the YS and BHS simples (Fig 6B).

### Screening cultivable protease-producing bacteria from the three China Seas

Compositional analysis of microbial communities also found that a large number of protease-producing microorganisms exist in the marine environment. Many cultivable protease-producing bacteria belong to the phylum Proteobacteria, especially the class Gammaproteobacteria [23]. In this study, there were 87 morphologically different colonies with hydrolytic zones that were purified, and the 16S rDNA sequences were submitted to the NCBI database (S1 Table).

The 16S rRNA gene sequences (>1,400 bp) obtained from all 87 isolates were classified into 12 genera (Table 1). Except for a single isolate (WH05-4) from the YS belonging to *Flavobacterium* in the Bacteroidetes phylum, the rest of the isolates were classified into 11 genera within the phyla Proteobacteria and Firmicutes. These included *Pseudoalteromonas*, *Vibrio*, *Pseudomonas*, *Alteromonas*, *Psychrobacter*, *Photobacterium*, *Colwellia*, *Marinobacter*, *Sulfitobacter* and *Ruegeria* in Proteobacteria, and *Bacillus* in Firmicutes. *Pseudoalteromonas* and *Bacillus* were isolated in all three sea areas but were most dominant in the BHS, and *Alteromonas* (23.8%) was the predominant genus in the YS. Furthermore, the protease-producing bacteria isolated from the YS belonged to 7 genera, which showed higher diversity than those isolated from the SCS and BHS. Only four genera were identified from the BHS, representing the least diverse community among three sea areas. The study of cultivable protease-producing marine bacteria will benefit the future utilization of marine microbial resources and the production of traditional fermented foods, industrial enzymes and bioactive peptides.

**Table 1:** Distribution of cultivable protease-producing bacteria in three sea areas.

Phyla	Genera	Sea areas		
		SCS	YS	BHS
*Proteobacteria*	*Pseudoalteromonas*	5	4	41
	*Vibrio*	3	2	0
	*Pseudomonas*	1	0	0
	*Alteromonas*	0	5	0
	*Psychrobacter*	1	0	0
	*Photobacterium*	1	0	0
	*Colwellia*	0	0	2
	*Marinobacter*	0	1	0
	*Sulfitobacter*	0	0	1
	*Ruegeria*	0	1	0
*Firmicutes*	*Bacillus*	2	7	9
*Bacteroidetes*	*Flavobacterium*	0	1	0

### Analysis of environmental factors in specific sampling sites

**Fig 7:**
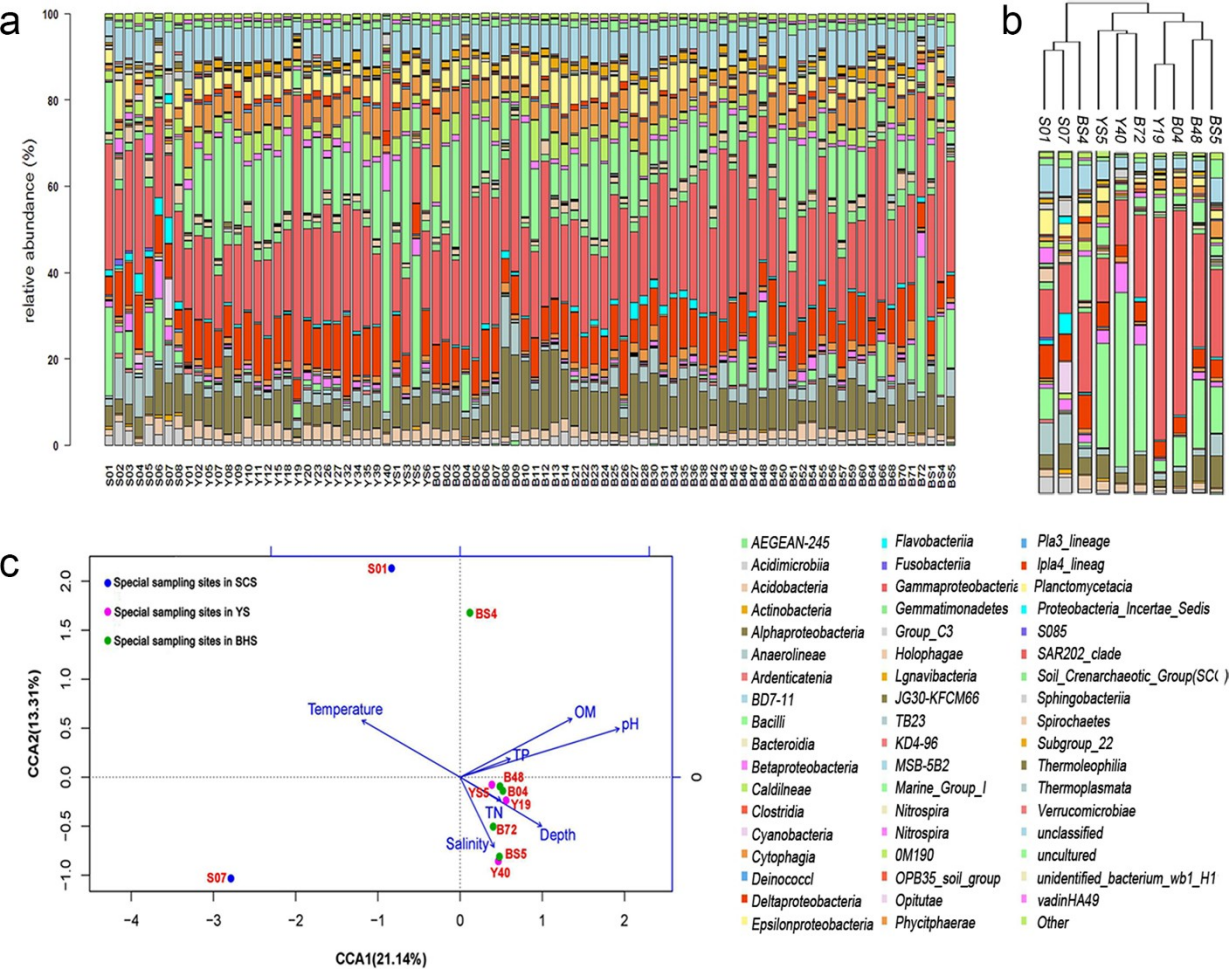
(a) Histogram method for determining the relative abundance of microorganisms at 86 sampling sites in the three China seas at the class level. (b) Relative abundance of microorganisms at ten sites of particular interest. (c) Canonical correspondence analysis (CCA) biplot showing the OTU-level community structure of chosen sampling sites and the physicochemical characteristics.

The results of the cluster analysis (Fig 5A) showed that there are ten sites of interest (S01, S07, Y19, Y40, YS5, B04, B48, B72, BS4 and BS5) out of all the sampling sites. As showed in Fig 7A and 7B, the microbial community structures in some of the sampling sites were significantly different from those of the other sampling sites at the class level. To determine what caused the distinct microbial compositions in these sampling sites the environmental parameters were tested. The chemical features of sediments varied from sample to sample (Table 2). The pH of the sediments ranged from 6.65 to 7.50, and all sediments were weakly alkaline, except S07 (pH = 6.65). The salinity ranged from 3.021‰ to 8.913‰. Some samples from the BHS, such as B48, B72 and BS5, contained higher concentrations of salt (over 8.171). The concentrations of organic matter ranged from 1.627 to 2.652 μg/g. The concentrations of total phosphorus and total nitrogen varied from 0.045 to 11.611 μg/g and 89.524 to 941.905 μg/g, respectively, in the different sediment samples; this indicated that the concentration of nitrogen in the sediment was much higher than that of the phosphorus.

**Table 2:** Enviromental factors of the sediment and overlying water samples.

Sampe ID	TP (μg/g)	TN (μg/g)	OM (μg/g)	pH	Depth (m)	Temperature (°C)	Salinity (PSU*)
S01	1.665	108.571	2.297	7.25	11.275	31.283	4.238
S07	1.665	218.095	1.627	6.65	11.163	31.541	4.752
Y19	3.600	89.524	2.652	7.45	25.035	30.517	4.028
Y40	0.045	332.381	1.903	7.68	18.031	31.280	4.028
YS5	11.611	270.476	2.652	7.28	18.031	31.280	4.028
B04	9.676	275.238	2.455	7.46	15.197	30.997	3.021
B48	1.980	208.571	2.573	7.34	11.485	30.264	8.913
B72	4.185	213.333	2.652	7.16	13.045	30.076	8.171
BS4	4.230	603.810	2.218	7.50	12.930	31.612	4.027
BS5	0.405	941.905	1.824	7.33	12.296	31.079	8.796

TP, total phosphorus; TN, total nitrogen; OM, organic matter.

* The practical salinity units (PSU) represented the salinity standard for no unit dimension in oceanography, and usually expressed in ‰.

CCA was used to study the correlations between sediment microbial groups and environmental variables based on the ten sediment samples. Environmental variables in the first two CCA dimensions explained 21.14% (CCA1) and 13.31% (CCA2) of the variance in the sediment microbial communities, respectively (Fig 7C). Among the tested physicochemical factors, pH (*P* = 0.034) showed a significant impact on the sediment community structure. Many studies have proved that marine microbial diversity and composition are sensitive to ocean pH shift [24, 25]. Krause et al. found that Gammaproteobacteria, Flavobacteriaceae, Rhodobacteraceae, Campylobacteraceae were as phylogenetic groups responding remarkably to differences in pH and the order Flavobacteriaceae was one of the most dominant bacterial groups found at low pH sites [26]. Similar to the result of Krause, our study also showed that Flavobacteriaceae was the predominant order (2.92%) in the low pH site (Site S07, pH 6.65) (Fig 7). The OM content (*P* = 0.134), depth (*P* = 0.271) and temperature (*P* = 0.218) had a moderate impact on the sediment community structure. The microbial community retrieved from different samples responded differently to environmental factors, and the responses were closely related to the specific physicochemical properties of the sediments. In particular, the microbial communites in the S01 and S07 sampling sites were positively correlated with temperature while the communities at the Y19, Y40, YS5, BS5, B04, B48 and B72 sampling sites showed significant correlations with depth and the concentration of the TN. In addition, the similarity of microbial communities in eight sites from the YS and BHS was higher than that of the two sites from the SCS, and the two sites of the SCS (S01 and S07) separated far from each other due to geographical location, which was consistent with the cluster analysis chart (Fig 5A).

## Discussion

Investigating the diversity of microorganisms and protease-producing bacteria in sediments is essential for understanding the ecological role of microorganisms in these habitats; thus, research examining how global biogeochemical cycles function, in which protease-producing bacteria are indispensable participants, is vital to the understanding of organic nitrogen decomposition and recycling processes in marine ecosystems. However, information on the bacterial diversity and the biogeochemical cycles present in most regions is lacking, particularly in Chinese marginal seas where there is intense nitrogen biogeochemical cycling [1, 13]. In this study, using next-generation sequencing technology, the diversity of bacteria in three offshore seas in China were systematically analyzed, collecting a total of 86 sediments from the BHS, YS and SCS. The microbial community composition in different international sea areas showed different phyla distributions. As shown in Table 3, Proteobacteria, as the dominant phylum appeared widely in most marine sediments, which agreed with the results of our study. Many other major phyla mentioned in other marine sediments were abundant in the three China seas as well, such as Planctomycetes, Acidobacteria, Firmicutes, Chloroflexi, Bacteroidetes, Actinobacteria and Gemmatimonadetes, which revealed a similar microbial community composition between the three China seas and other seas.

**Table 3:** Dominant and major microbial diversities and community compositions in different international sea areas from published literatures.

Site	Time	Dominant phyla	Major phyla	Data sources	References
Jeju Island	2016	*Proteobacteria*, *Bacteroidetes*	*Actinobacteria*, *Acidobacteria*, *Firmicutes*	Microbial Community Composition in the Marine Sediments of Jeju Island: Next-Generation Sequencing Surveys	[5]
Mid-Atlantic Ridge	2015	*Proteobacteria*, *Planctomycetes*	*Acidobacteria*, *Bacteroidetes*, *Gemmatimonadetes*, *Nitrospirae*	Microbial diversity in deep-sea sediments from the Menez Gwen hydrothermal vent system of the Mid-Atlantic Ridge	[27]
Eastern Mediterranean, Turkey	2015	*Proteobacteria*	*Acidobacteria*, *Firmicutes*, *Bacteroidetes*	Seasonal abundance and diversity of culturable heterotrophic bacteria in relation to environmental factors in the Gulf of Antalya, Eastern Mediterranean, Turkey	[28]
Changjiang Estuary and in the East China Sea	2015	*Proteobacteria*, *Bacteroidetes*, *Planctomycetes*	*Acidobacteria*, *Chloroflexi*, *Actinobacteria*, *Gemmatimonadetes*	Bacterial diversity in the surface sediments of the hypoxic zone near the Changjiang Estuary and in the East China Sea	[10]
New Brunswick, Canada	2014	*Proteobacteria*, *Bacteroidetes*	*Actinobacteria*, *Verrucomicrobia*, *Acidobacteria*	Bioprospecting from Marine Sediments of New Brunswick, Canada: Exploring the Relationship between Total Bacterial Diversity and *Actinobacteria* Diversity	[29]
The East China Sea	2013	*Proteobacteria*, *Chloroflexi*, *Planctomycetes*	*Acidobacteria*, *Actinobacteria*, *Bacteroidetes*	Bacterial diversity in surface layer of sediment in East China Sea	[30]
Yam O Wan in Hong Kong	2012	*Proteobacteria*	*Planctomycetes*, *Acidobacteria*, *Chloroflexi*, *Verrucomicrobia*, *Nitrospira*, *Firmicutes*	Comparison of the Levels of Bacterial Diversity in Freshwater, Intertidal Wetland, and Marine Sediments by Using Millions of Illumina Tags	[31]
Arctic	2012	*Proteobacteria*	*Actinobacteria*, *Aciodobacteria*, *Bacteroidetes*, *Nitrospirae*, *Firmicutes*, *Deinococcus*	The Investigation on Microbial Diversity of Artic Deep Sea Sediments	[32]
Laizhou Bay Sediments, Bohai Sea, China	2016	*Proteobacteria*	*Firmicutes*, *Actinobacteria*, *Bacteroidetes*,	Screening of Protease-Producing Bacteria from Sediment Samples	[33]

At the phylum level, Proteobacteria and Planctomycetes were the most dominant phyla in the three China Seas; bacteria in these phyla play an important role in the marine nitrogen cycle. Proteobacteria play a vital role in degrading sedimentary organic nitrogen [7, 9], while Planctomycetes are proposed to exhibit unique biogeochemical properties like anaerobic ammonium oxidation [34], methane oxidation [35], and participation in carbon recycling [36]. Chloroflexi and Cyanobacteria appeared to be more aboundant in the SCS than in the BHS and YS. As two types of photoautotrophic bacteria, Chloroflexi and Cyanobacteria have the capacity for nitrogen and carbon fixation and play crucial roles in the nitrogen and carbon cycles in marine ecosystems [37]. Moreover, Firmicutes was detected in all three China seas. Antonella et al. studied the bacterial community structure of the sediment from a high Arctic fjord and found that the high relative abundance of Firmicutes (up to 58%) retrieved in anoxic marine sediments and the predominance of Proteobacteria, in cooccurrence with the Bacteroidetes, Firmicutes and Actinobacteria, suggested the presence of nutrients for heterotrophic bacterial growth along the habitat [38]. Many researches have reported that the offshore environments were much affected by human beings [9,13]. We also analyzed all offshore sampling sites at the family level in the three China seas, and similar to previous research, our study found that Proteobacteria and Firmicutes accounted for a large proportion of the identified bacteria, and they were all chemotrophic bacteria in the three China seas. Proteobacteria (especially Alphaproteobacteria and Gammaproteobacteria) are the most important component of the offshore microbiome. This might be due to the environmental eutrophication caused by human activities [39, 40]. Intriguingly, as mentioned above, the YS cluster II had fewer chemoheterotrophic and aerobic chemoheterotrophy microorganisms (Fig 5B). These sites in the YS cluster II are far from the inland (Fig 1) where they might be less affected by human activity. Although sites Y27, Y39 and Y40 were also far from the inland, these sites are close to the Changjiang estuary and are affected by international shipping. Furthermore, Firmicutes was enriched in nutrient-rich areas, also revealing that the degree of eutrophication in the SCS was relatively severe. A large number of microorganisms reliant on nutrition were found in the SCS mainly because sampling sites were close to the inland and were influenced by frequent human activities; for example, harmful algae grew excessively when the land-based nutrient content of seawater increased steadily [39]. On the other hand, Hydrogenedentes was more abundant in the BHS and YS than the SCS. Hydrogenedentes is often associated with methanogenic environments [40]. Nobu et al. reported that Hydrogenedentes was a lipolytic glycerol degrader. They found that in area with low Hydrogenedentes population abundance (0.8%), the hydrogenase gene expression level was strikingly high (10.1% of bacterial transcriptome), which suggested that Hydrogenedentes lipolysis and glycerol degradation is an important component of the terephthalate degrading community carbon flux [41]. Besides, The Bohai Sea and the Yellow Sea have enormous reserves of natural gas fields. The investigation of the dissolved methane distributions in 2012 showed that in the Bohai Sea, episodic oil and gas spill events increased the surface methane concentration by up to 4.7 times and raised the local methane outgassing rate by up to 14.6 times [42]. Thus, the abundance of Hydrogenedentes in the BHS and YS might be caused by the episodic gas spill events and the diversity of the Hydrogenedentes might be used as an environmental remediation index of oil field pollution.

Overall, among microbial communities, some microbial groups showed a specific pattern in the three different seas. For instance, the family *Anaerolineaceae* was significantly dominant in the sample sites S01, S02, S03, S04 and S07 in the South China Sea compared with the YS and the BHS. Previous research on the bacterial community in permafrost soils along the China-Russia crude oil pipeline also found that *Anaerolineaceae* were very common, accounting for 8.27% of the total libraries [43]. This indicated that the members of Anaerolineaceae were able to live in the environment with oil pollution. This phenomenon was also observed in this study. The reason for the high abundance of Anaerolineaceae might be due to the oil gas fields near the South China Sea [13]. Previous studies reported that Anaerolineaceae showed the potential for hydrocarbon degradation. A recent study revealed that Anaerolineaceae might be involved in the activation and biodegradation of *n*-octane and *n*-decane or play a role in scavenging metabolic intermediates of methanogenic biodegradation [44]. The families Streptococcaceae and Flavobacteriales were also dominant among the sample sites of the South China Sea. The reason for the relatively higher abundance of Streptococcaceae and Flavobacteriales could be the nearby fish farm. Both of these bacteria are thought to be a typical kind of pathogenic bacteria in fish [45]. Many studies have demonstrated that differences in microbial composition in different areas are closely related to environmental factors [46]. In the BHS, it was possible that the major microbial groups were affected by environmental factors, especially total nitrogen. It was clear that the families Pseudoalteromonadaceae and Alteromonadales were abundant in the BHS, especially in offshore areas, such as B64, B66, B71, B72 and BS5. Bacillaceae were also the major group at the B72 and BS5 sites. Most of the members of the families above are thought to play important roles in the biodegradation of organic nitrogen. According to the environmental factors, the total nitrogen content was commonly high in these BHS sample sites (208.571–941.905 μg/g) especially for the sites BS4 and BS5. Intriguingly, the indicator of bacterial groups in sediments of BHS are Proteobacteria, Gammaproteobacteria, Alphaproteobacteria, Pseudoalteromonadaceae and Alteromonadales (Fig 3B). These bacteria are typical heterotrophic bacteria that play important roles in the degradation of marine organic nitrogen [4]. Since the Bohai Sea is located within the arms of the Liaodong and Shandong Peninsulas, the BHS is considered as an area affected by human activity. Several rivers empty into the Bohai Sea, meaning that the municipal wastewater from surrounding cities is also discharged into the sea and causes higher total nitrogen content.

Microorganisms reliant on nutrition are thought to play an important role in nitrogen decomposition, especially protease-producing bacteria. These decomposers represent an excellent source of proteases and are the main source of production. Since the ocean environment has distinctive features compared with terrestrial environments, such as a lower temperature, higher salinity and greater oligotrophy, proteases from marine bacteria commonly display stronger cold- and salt- adaption and possess higher catalytic activities [47]. In a previous study, Ye et al. showed that most cultivable bacteria presented in the East China Sea are Proteobacteria, Chloroflexi, and Planctomycetacia. Similarly, our research also found that Proteobacteria, Planctomycetacia and Chloroflexi were the most abundant bacteria present in the three studied China offshore seas [10]. Zhang et al. found that *Photobacterium*, *Bacillus*, and *Vibrio* were the richest cultivated protease-producing bacteria in Jiaozhou Bay [7]. However, the results of screening cultivable protease-producing bacteria from three China Seas showed that *Pseudoalteromonas*, *Bacillus* and *Vibrio* were the richest cultivatable protease-producing bacteria, and *Pseudoalteromonas* was the most abundant. The existence of the genus *Photobacterium* was not detected. *Pseudoalteromonas* is a typical marine microorganism and widely exists in marine environments including deep sea, sea ice and vent habitats [6, 48]. Affected by ocean currents, *Pseudoalteromonas* might be rich in offshore sediments. A series of studies on marine bacterial proteases have been reported, indicating good prospects for future applications, such as the production of traditional fermented foods, and the preparation of bioactive peptides from low-valued protein resources, in addition to other applications developed by the industrial enzyme sector [49, 50].

Our research shows that human activities have a great impact on the diversity of microorganisms in China's offshore sea. Proteobacteria and Planctomycetes are the dominant phyla in the three China marginal seas (BHS, YS and SCS). Especially in BHS, the indicator of bacterial groups are Proteobacteria, Gammaproteobacteria, Alphaproteobacteria, Pseudoalteromonadaceae and Alteromonadales, which are typical heterotrophic bacteria that play important roles in the degradation of marine organic nitrogen. The research on protease-producing bacteria shows that the predominant protease-producing genera are *Pseudoalteromonas* and *Bacillus*. This study systematically investigated the composition of microbial communities in China's offshore seas, laying a theoretical basis for environmental protection and the sustainable development of marine microbial resources, especially for protease-producing bacteria.

## Supporting information

S1 Table Diversity of cultivable protease-producing bacteria in three China seas. (DOCX)
