# Supplementary material for: Diversity of the microbial community and cultivable protease-producing bacteria in the sediments of the Bohai Sea, Yellow Sea and South China Sea
Source: PLoS One. 2019 Apr 11;14(4):e0215328. doi: 10.1371/journal.pone.0215328 (PMC6459509; doi:10.1371/journal.pone.0215328)
Supplement: S1 Table — (DOCX) [file pone.0215328.s001.docx]

**Table S1** Diversity of cultivable protease-producing bacteria in three China seas.

|  | SeqId | Organism | Isolation-source | Country | note | GenBank accession numbers |
| --- | --- | --- | --- | --- | --- | --- |
| BHS | B10-4 | *Bacillus* sp. B10-4 | Bohai_Sea | China | pure_culture | MG388109 |
|  | B25-3 | *Bacillus* sp. B25-3 | Bohai_Sea | China | pure_culture | MG388113 |
|  | B27-2 | *Bacillus* sp. B27-2 | Bohai_Sea | China | pure_culture | MG388114 |
|  | B27-4 | *Bacillus* sp. B27-4 | Bohai_Sea | China | pure_culture | MG388116 |
|  | B28-1 | *Bacillus* sp. B28-1 | Bohai_Sea | China | pure_culture | MG388117 |
|  | B28-2 | *Bacillus* sp. B28-2 | Bohai_Sea | China | pure_culture | MG388118 |
|  | B36-2 | *Bacillus* sp. B36-2 | Bohai_Sea | China | pure_culture | MG388125 |
|  | B47-7 | *Bacillus* sp. B47-7 | Bohai_Sea | China | pure_culture | MG388141 |
|  | ZB23-3 | *Bacillus* sp. ZB23-3 | Bohai_Sea | China | pure_culture | MG388172 |
|  | B47-5 | *Colwellia* sp. B47-5 | Bohai_Sea | China | pure_culture | MG388139 |
|  | B61-1 | *Colwellia* sp. B61-1 | Bohai_Sea | China | pure_culture | MG388144 |
|  | B06-1 | *Pseudoalteromonas* sp. B06-1 | Bohai_Sea | China | pure_culture | MG388102 |
|  | B06-2 | *Pseudoalteromonas* sp. B06-2 | Bohai_Sea | China | pure_culture | MG388103 |
|  | B06-3 | *Pseudoalteromonas* sp. B06-3 | Bohai_Sea | China | pure_culture | MG388104 |
|  | B08-1 | *Pseudoalteromonas* sp. B08-1 | Bohai_Sea | China | pure_culture | MG388105 |
|  | B08-2 | *Pseudoalteromonas* sp. B08-2 | Bohai_Sea | China | pure_culture | MG388106 |
|  | B08-3 | *Pseudoalteromonas* sp. B08-3 | Bohai_Sea | China | pure_culture | MG388107 |
|  | B08-4 | *Pseudoalteromonas* sp. B08-4 | Bohai_Sea | China | pure_culture | MG388108 |
|  | B22-1 | *Pseudoalteromonas* sp. B22-1 | Bohai_Sea | China | pure_culture | MG388110 |
|  | B25-1 | *Pseudoalteromonas* sp. B25-1 | Bohai_Sea | China | pure_culture | MG388111 |
|  | B25-2 | *Pseudoalteromonas* sp. B25-2 | Bohai_Sea | China | pure_culture | MG388112 |
|  | B27-3 | *Pseudoalteromonas* sp. B27-3 | Bohai_Sea | China | pure_culture | MG388115 |
|  | B28-3 | *Pseudoalteromonas* sp. B28-3 | Bohai_Sea | China | pure_culture | MG388119 |
|  | B28-4 | *Pseudoalteromonas* sp. B28-4 | Bohai_Sea | China | pure_culture | MG388120 |
|  | B35-3 | *Pseudoalteromonas* sp. B35-3 | Bohai_Sea | China | pure_culture | MG388122 |
|  | B35-4 | *Pseudoalteromonas* sp. B35-4 | Bohai_Sea | China | pure_culture | MG388123 |
|  | B36-1 | *Pseudoalteromonas* sp. B36-1 | Bohai_Sea | China | pure_culture | MG388124 |
|  | B36-3 | *Pseudoalteromonas* sp. B36-3 | Bohai_Sea | China | pure_culture | MG388126 |
|  | B36-4 | *Pseudoalteromonas* sp. B36-4 | Bohai_Sea | China | pure_culture | MG388127 |
|  | B36-5 | *Pseudoalteromonas* sp. B36-5 | Bohai_Sea | China | pure_culture | MG388128 |
|  | B40-3 | *Pseudoalteromonas* sp. B40-3 | Bohai_Sea | China | pure_culture | MG388129 |
|  | B40-4 | *Pseudoalteromonas* sp. B40-4 | Bohai_Sea | China | pure_culture | MG388130 |
|  | B40-5 | *Pseudoalteromonas* sp. B40-5 | Bohai_Sea | China | pure_culture | MG388131 |
|  | B43-1 | *Pseudoalteromonas* sp. B43-1 | Bohai_Sea | China | pure_culture | MG388132 |
|  | B43-2 | *Pseudoalteromonas* sp. B43-2 | Bohai_Sea | China | pure_culture | MG388133 |
|  | B43-3 | *Pseudoalteromonas* sp. B43-3 | Bohai_Sea | China | pure_culture | MG388134 |
|  | B43-4 | *Pseudoalteromonas* sp. B43-4 | Bohai_Sea | China | pure_culture | MG388135 |
|  | B47-1 | *Pseudoalteromonas* sp. B47-1 | Bohai_Sea | China | pure_culture | MG388136 |
|  | B47-3 | *Pseudoalteromonas* sp. B47-3 | Bohai_Sea | China | pure_culture | MG388137 |
|  | B47-4 | *Pseudoalteromonas* sp. B47-4 | Bohai_Sea | China | pure_culture | MG388138 |
|  | B47-6 | *Pseudoalteromonas* sp. B47-6 | Bohai_Sea | China | pure_culture | MG388140 |
|  | B53-1 | *Pseudoalteromonas* sp. B53-1 | Bohai_Sea | China | pure_culture | MG388142 |
|  | B53-3 | *Pseudoalteromonas* sp. B53-3 | Bohai_Sea | China | pure_culture | MG388143 |
|  | B61-3 | *Pseudoalteromonas* sp. B61-3 | Bohai_Sea | China | pure_culture | MG388145 |
|  | B62-1 | *Pseudoalteromonas* sp. B62-1 | Bohai_Sea | China | pure_culture | MG388146 |
|  | B62-3 | *Pseudoalteromonas* sp. B62-3 | Bohai_Sea | China | pure_culture | MG388147 |
|  | B66-3 | *Pseudoalteromonas* sp. B66-3 | Bohai_Sea | China | pure_culture | MG388148 |
|  | B68-3 | *Pseudoalteromonas* sp. B68-3 | Bohai_Sea | China | pure_culture | MG388149 |
|  | ZB23-2 | *Pseudoalteromonas* sp. ZB23-2 | Bohai_Sea | China | pure_culture | MG388171 |
|  | ZB23-4 | *Pseudoalteromonas* sp. ZB23-4 | Bohai_Sea | China | pure_culture | MG388173 |
|  | ZB23-5 | *Pseudoalteromonas* sp. ZB23-5 | Bohai_Sea | China | pure_culture | MG388174 |
|  | ZB23-6 | *Pseudoalteromonas* sp. ZB23-6 | Bohai_Sea | China | pure_culture | MG388175 |
|  | B28-5 | *Sulfitobacter* sp. B28-5 | Bohai_Sea | China | pure_culture | MG388121 |
| YS | WH09-4 | *Alteromonas* sp. WH09-4 | Yelllow_Sea | China | pure_culture | MG388158 |
|  | WH10-2 | *Alteromonas* sp. WH10-2 | Yelllow_Sea | China | pure_culture | MG388159 |
|  | WH14-2 | *Alteromonas* sp. WH14-2 | Yelllow_Sea | China | pure_culture | MG388163 |
|  | WH16-2 | *Alteromonas* sp. WH16-2 | Yelllow_Sea | China | pure_culture | MG388165 |
|  | WH22-3 | *Alteromonas* sp. WH22-3 | Yelllow_Sea | China | pure_culture | MG388168 |
|  | WH05-3 | *Bacillus* sp. WH05-3 | Yelllow_Sea | China | pure_culture | MG388151 |
|  | WH09-3 | *Bacillus* sp. WH09-3 | Yelllow_Sea | China | pure_culture | MG388157 |
|  | WH13-1 | *Bacillus* sp. WH13-1 | Yelllow_Sea | China | pure_culture | MG388160 |
|  | WH13-3 | *Bacillus* sp. WH13-3 | Yelllow_Sea | China | pure_culture | MG388161 |
|  | WH13-4 | *Bacillus* sp. WH13-4 | Yelllow_Sea | China | pure_culture | MG388162 |
|  | WH21-3 | *Bacillus* sp. WH21-3 | Yelllow_Sea | China | pure_culture | MG388167 |
|  | WH31-2 | *Bacillus* sp. WH31-2 | Yelllow_Sea | China | pure_culture | MG388170 |
|  | WH05-4 | *Flavobacterium* sp. WH05-4 | Yelllow_Sea | China | pure_culture | MG388152 |
|  | WH16-3 | *Marinobacter* sp. WH16-3 | Yelllow_Sea | China | pure_culture | MG388166 |
|  | WH05-1 | *Pseudoalteromonas* sp. WH05-1 | Yelllow_Sea | China | pure_culture | MG388150 |
|  | WH06-1 | *Pseudoalteromonas* sp. WH06-1 | Yelllow_Sea | China | pure_culture | MG388153 |
|  | WH06-2 | *Pseudoalteromonas* sp. WH06-2 | Yelllow_Sea | China | pure_culture | MG388154 |
|  | WH16-1 | *Pseudoalteromonas* sp. WH16-1 | Yelllow_Sea | China | pure_culture | MG388164 |
|  | WH31-1 | *Ruegeria* sp. WH31-1 | Yelllow_Sea | China | pure_culture | MG388169 |
|  | WH06-3 | *Vibrio* sp. WH06-3 | Yelllow_Sea | China | pure_culture | MG388155 |
|  | WH09-1 | *Vibrio* sp. WH09-1 | Yelllow_Sea | China | pure_culture | MG388156 |
| SCS | SXN2 | *Bacillus* sp. SXN2 | South China Sea | China | pure_culture | MG383480 |
|  | SXS3-1 | *Bacillus* sp. SXS3-1 | South China Sea | China | pure_culture | MG383482 |
|  | SJS7 | *Photobacterium* SJS7 | South China Sea | China | pure_culture | MG383477 |
|  | SQN2-1 | *Pseudoalteromonas* sp. SQN2-1 | South China Sea | China | pure_culture | MG383472 |
|  | SBN2-1 | *Pseudoalteromonas* sp. SBN2-1 | South China Sea | China | pure_culture | MG383475 |
|  | SHK | *Pseudoalteromonas* sp. SHK | South China Sea | China | pure_culture | MG383476 |
|  | SJS4 | *Pseudoalteromonas* sp. SJS4 | South China Sea | China | pure_culture | MG383478 |
|  | SJS4-1 | *Pseudoalteromonas* sp. SJS4-1 | South China Sea | China | pure_culture | MG383479 |
|  | SXS1 | *Pseudomonas* sp. SXS1 | South China Sea | China | pure_culture | MG383481 |
|  | SXS3-2 | *Psychrobacter* sp. SXS3-2 | South China Sea | China | pure_culture | MG383483 |
|  | SBN | *Vibrio* sp. SBN | South China Sea | China | pure_culture | MG383473 |
|  | SBN1 | *Vibrio* sp. SBN1 | South China Sea | China | pure_culture | MG383474 |
|  | GN2-2 | *Vibrio* sp. GN2-2 | South_China_Sea | China | pure_culture | MG388176 |
